# Effect of a Supplementation with Two *Quelites* on Urinary Excretion of Arsenic in Adolescents Exposed to Water Contaminated with the Metalloid in a Community in the State of Guanajuato, Mexico

**DOI:** 10.3390/nu12010098

**Published:** 2019-12-30

**Authors:** Yair Olovaldo Santiago-Saenz, Rebeca Monroy-Torres, Diana Olivia Rocha-Amador, Alma Delia Hernández-Fuentes

**Affiliations:** 1 Área Académica de Ingeniería Agroindustrial e Ingeniería en Alimentos, Instituto de Ciencias Agropecuarias, Universidad Autónoma del Estado de Hidalgo, Tulancingo 43600, Mexico; y.sant.sanz@gmail.com; 2 Departamento de Medicina y Nutrición, División de Ciencias de la Salud, Universidad de Guanajuato, León 37670, Mexico; 3 Departamento de Farmacia, División de Ciencias Naturales y Exactas, Universidad de Guanajuato, Guanajuato 36050, Mexico; olivia2000_mx@hotmail.com

**Keywords:** *quelites*, supplementation, arsenic

## Abstract

*Quelites* are Mexican wild plants, reported as excellent sources of nutritional compounds such as amino acids (serine, glycine, and cysteine), minerals (Mg, Fe, and Zn), and phytochemicals, as phenolic acids (chlorogenic acid) and flavonoids (phloridzin and naringenin); on the other hand, high biological activity has been shown in these compounds. This work aimed to evaluate the effect of a supplementation with two endemic *quelites* of Mexico (*Chenopodium berlandieri* L. and *Portulaca Oleracea* L.); in addition to supplementation, a nutritional intervention was performed; the biomarkers of hemoglobin (Hb), urinary malondialdehyde (UMDA), and urinary arsenic (UAs) were measured in adolescents exposed to arsenic. A clinical intervention study was conducted in 27 adolescents ages 11 to 12 years for 4 weeks. Weekly anthropometric and dietary evaluations were carried out, as well as the concentration of Hb; the UMDA and UAs were performed by plate-based colorimetric measurement and atomic absorption spectrophotometry with the hydrides generation system, respectively. The results showed that UMDA concentrations had a significant improvement in the supplemented group (SG) vs. control group (CG) (SG = 1.59 ± 0.89 µM/g creatinine vs. CG = 2.90 ± 0.56 µM/g creatinine) in the second week of intervention; on the other hand, the supplemented group showed an increase in Hb levels (15.12 ± 0.99 g/dL) in the same week; finally after the second week, an increase in UAs levels was observed significantly compared to the baseline value (Baseline: 56.85; Week 2: 2.02 µg/g creatinine). Therefore, the results show that the mixture of *quelites* (a rich source of phytochemicals and nutrients) improved hemoglobin and UMDA levels, and urinary arsenic excretion from the second week in the exposed population.

## 1. Introduction

Arsenic (As) is an element that is naturally distributed on the surface of the earth’s crust, it can be filtered to groundwater reserves, due to soil drilling, water movements, and when there is mining activity. In other cases, depending on the composition of the rock, water can dissolve and drag these elements, producing natural pollution and increasing the concentration in ground and surface waters [1,2], which become the main source of drinking water in rural areas. There are many other types of pollution of As, including chemical pollution into bodies of water and soil through improper disposal practices and agricultural activities, due for use of pesticides and fertilizers, among other factors that compromise human health.

In countries such as Thailand, India, China, Argentina, and Mexico, concentrations of As have been found in ranges from 0.5 to 5000 µg/L [3,4]. As for Mexico, the highest concentrations of As are found in the northeast and central parts of the country [5], generally in arid and semi-arid areas, including Coahuila, Zacatecas, Hidalgo, San Luis Potosí, and Guanajuato [6]. Particularly in the state of Guanajuato, in the northeast region, given its location in the vicinity of three geological provinces, combined with volcanic activity alternated with periods of intense erosion and sedimentation, give rise to different dissolved elements [1,7]. But in the central area of the state of Guanajuato, since 2004, they have been identified in several communities with high levels of As in water [8].

According to the World Health Organization (WHO) [9], As, due to its ubiquity characteristic, is classified as one of the chemicals of greatest concern worldwide, which, if not handled properly, can be very dangerous for the environment and health. Chronic exposure to As can cause hyperkeratosis, dermatitis, respiratory diseases, cancer, adverse thyroid effects, and neurotoxicity [8,9,10,11].

Few studies have shown that a high intake of antioxidants and fructooligosaccharides (FOS) identified in the Mexican diet foods such as beans, lentils, bananas, and onions can prevent or reverse some of the effects involved in the development of cancer and cardiovascular diseases and chronic, caused by this pollutant, by avoiding bioaccumulate in the organism and thus an improvement in the excretion in the urine [12,13]; therefore, the consumption of foods rich in phytochemicals with adequate nutritional contributions and with high antioxidant capacity, has been considered a strategy to reduce the effects of this element when people have acute or chronic exposure, by providing the requirements of various nutrients such as cysteine, methionine, vitamin C [14,15], and phenolic compounds [16,17,18], essential for arsenic detoxification [19], to increase antiradical effects (ROS), and reduce the production of malondialdehyde (MDA) in the body [17,18].

Mexico has a wide variety of wild edible plants found in rural areas referred to as *quelites* [20]; between them, we can differentiate two important species, *Chenopodium berlandieri* L. and *Portulaca oleraceae* L., which naturally grow in abundance in mountains, flat fields, or near corn or bean crops of Mexican territory [21]. The leaves and stems of these species have a high nutritional contribution (dietary fiber, proteins, minerals) and bioactive compounds (carotenoids, chlorophyll, phenolic acids, and flavonoids) [22], as well as a high antioxidant capacity and provide biological effects of interest in human health [21,22]. Recent studies have found excellent results when using plants that belong to the genera of *Chenopodium* spp., reporting a reduction in MDA and increased activity of antioxidant enzymes in rats induced with toxic agents [23] or decreasing levels of lipid peroxidation in lines of cancer cells [24]. Finally, when using *Portulaca* spp. in animal models, the potential effect of its bioactive compounds on the integrity of the CNS (Central Nervous System) [25], hepatoprotective capacity [26], and the reduction of damage by stress mechanisms against external factors such as pollutants (rotenone) [27] have been observed.

In Mexico, there is evidence that shows that the administration of supplements that incorporate vegetables such as broccoli and fruits such as grapes, as well as minerals and vitamins essential for the detoxification process [14,15,16,17,18,19], promote better arsenic excretion and improve the nutritional status, in this case, by inducing an increase in hemoglobin (Hb) levels of participants [28]. However, given these bases and the nutritional contribution of these *quelites*, the aim of this research was to evaluate the effect of a supplement made with two novelty ingredients (*Portulaca oleraceae* L. and *Chenopodium berlandieri* L.), economical, accessible, and with sufficient evidence of its antioxidant and nutritional potential, on hemoglobin (Hb), urinary arsenic excretion (UAs), and urinary malondialdehyde (UMDA) concentration, which will be biomarkers of interest to know the positive effect of this supplement provided in adolescents from a rural area in Guanajuato, Mexico, exposed to arsenic.

## 2. Materials and Methods

### 2.1. Reagents and Equipments

Nitric acid, perchloric acid, sodium borohydride, potassium iodide, sodium hydroxide, hydrochloric acid, and ascorbic acid were purchased from J.T Baker (Avantor Performance Materials, Ecatepec, Estado de Mexico, Mexico) and Fermont (Rye S.A de C.V, Tlalnepantla, Estado de Mexico, Mexico). The microcuvettes for hemoglobin determination (Hb-201) were purchased from HemoCue (HemoCue, Ciudad de Mexico, Mexico). The TBARS assay kit (MDA) was purchased from Cayman Chemical (Cayman Chemical, Ann Arbor, MI, USA). The arsenic standard was purchased from Agilent (Agilent, Santa Clara, CA, USA). The quality control for As (ClinChek^®^-urine control lyophilised for trace elements, level II) was purchased from Iris Tech (IRIS Technologies International GmbH, Olathe, KS, USA). The concentration of arsenic (As) in drinking water with Arsenator^®^-Digital Arsenic Test Kit from Wagtech WTD, Palintest, CO, USA). All solutions were prepared using deionized water.

### 2.2. Intervention Design, Study Groups, and Acquisition of Data

**Figure 1:**
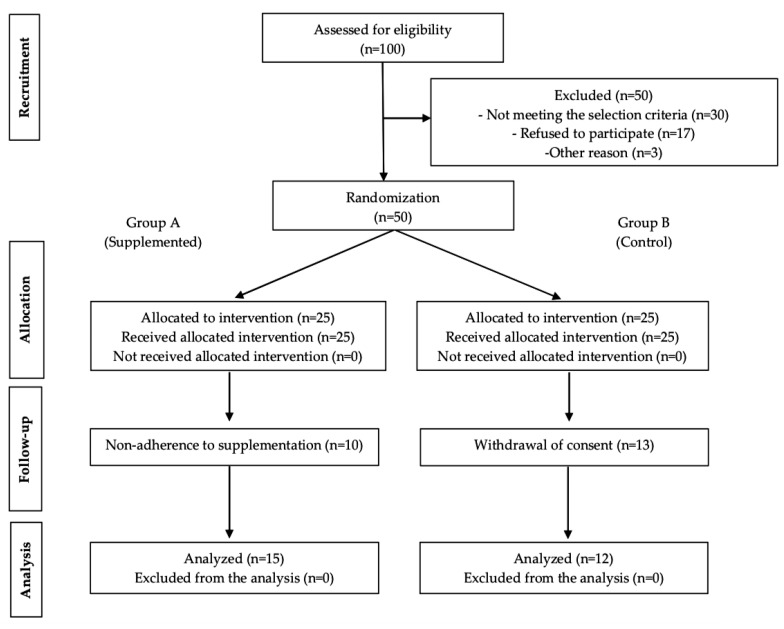
Flow diagram of patient progress through the phases of the randomized trial.

A clinical intervention study was conducted in 27 participants (13 men and 14 women) ages 11 to 12 years belonging to the rural area of Valencianita, Irapuato, in the state of Guanajuato, Mexico. It was a randomized study to assess the effect of supplementation on UAs excretion. The random assignment code was generated using a randomized block design with SAS^®^ (Cary, NC, USA). All participants were randomly assigned in into two groups referred to as control and supplemented (4 g of supplement). The details of the study are mentioned by section and in Figure 1.

#### 2.2.1. Eligibility Criteria

Inclusion criteria: High school students #117 of the community of Valencianita, Irapuato in the state of Guanajuato, men and women with a minimum residence of three years in the community, arsenic concentrations in drinking water above 25 μg/L (hydroarsenism) [29].

Exclusion criteria: Participants who are taking a supplement (trademarks of natural products), or drug (treatment or over-the-counter), participants with some type of kidney or liver damage confirmed by the clinical history, participants who did not want to join the study, and participants who provided a single urine sample or were evaluated only once.

#### 2.2.2. Pre-Selection Phase and Ethical Statement

Parents and adolescents received verbal and written information about the study, informed parental consent, and consent from the adolescents were obtained before they participated in the study. The study was free, anonymous, and voluntary, was conducted in accordance with the Declaration of Helsinki, and approved by the institutional research bioethics committee of the University of Guanajuato (Code-CIBIUG-P34-2018).

#### 2.2.3. Selection Phase and Clinical Intervention

Participants who met the inclusion criteria were incorporated into the study. It was confirmed that the adolescents included in the research did not have acute or chronic diseases, their liver function was normal, and they were not taking drugs or supplements before and during the intervention.

The adolescents were divided into two groups: the supplemented group with 15 participants (9 men and 6 women) and the control group with 12 participants (4 men and 8 women).

Before beginning treatment and during the intervention, parents and adolescents were informed of the importance of consuming treated and purified water to observe a better recovery process. In addition, a questionnaire was applied to the mothers of the participants for the acquisition of information concerning drinking water, and later these data were confirmed with the analysis of water samples consumed at the participants’ homes and provided by the mothers during the intervention.

The clinical intervention was carried out according to Monroy-Torres et al. [28]. A detailed nutritional history was obtained for each child and a physical examination was performed, which included anthropometric parameters.

### 2.3. Supplement of Quelites

In this research a powder supplement made with two endemic vegetable ingredients from Mexico was used; these plants contained, in dietary supplement, belong to the species of *Chenopodium berlandieri* L. and *Portulaca Oleraceae* L. In addition, each species was characterized [22], and also the nutritional and functional evaluation of the supplement was performed [30]; the research considered the nutritional profile [macronutrients and micronutrients (minerals and amino acids)] and antioxidant properties (antioxidant compounds and antioxidant activity) [30].

### 2.4. Dietary Supplementation

Parents and adolescents belonged to the supplemented group were informed about storage and intake of the supplement before the beginning of the study; two units of 60 g each of supplement were delivered to the parents for the total period of the intervention. The dose of the treatment was 4 g per day for 4 consecutive weeks (November 2018) and with a single dose 1 h before the intake of any food during morning hours, considering taking it at the same time every day; on the other hand, no placebo was administered to the control group because of the social and cultural factors previously identified in the rural community. The adolescents included in this work were not provided an orientation about an adequate diet, allowing them to maintain their diet as usual during the study. The supplement intake monitoring was evaluated by adherence questionnaires once a week at the school and by telephone two days per week. At the end of the study, the participants were asked to return any pouches not consumed. The dose of the supplement was selected by extrapolated calculations on the basis of a previous animal studies [23,27] and a previous clinical intervention [28].

### 2.5. Urine Sample

The urine samples were collected in polyethylene containers previously washed with HNO_3_ (10%) and being the first urine in the morning. These samples were requested from the participant minutes before the corresponding evaluation and stored in refrigeration at 4 °C until the end of the day indicators measurement. Later, samples were transported inside thermal containers. Before submitting the samples to the freezing process, a 1 mL aliquot of urine was extracted from each container and stored in previously labeled microtubes, which were taken to ultra-freezing (−76 °C) (Thermo-Scientific, 703, Outside, USA) for the subsequent MDA assay; the samples collected in the polyethylene containers were stored in freezing at −20 °C until the moment of analysis. The creatinine content of the samples was determined within the week of collection of each batch and according to the Jaffe reaction method [31]. The results of the urinary biomarkers were adjusted per grams of creatinine.

### 2.6. Anthropometric Assessment

Weight (Kg), fat mass (%), and lean body mass (%) were obtained with the impedance technique (bioimpedance analyzer, InBody^®^ R20); waist (cm) and abdominal (cm) circumferences (metal tape measure, Lufkin) were measured; also height (cm) (stadiometer, Seca^®^) of each participant was measured. The anthropometric measurements were obtained according to Lohman et al. [32]. With the weight and height, the body mass index (BMI) was calculated and they were classified in obesity, overweight, or normal weight; in addition, height-for-age (H/A) was obtained; WHO reference tables [33] and WHO Anthro Plus^®^ software were used for data interpretation. A BMI with thinness (malnutrition) with <−2 standard deviation (SD), normal between −2 to +1 (SD), overweight with >+1 (SD), and obesity with >+2 (SD) was considered. On the other hand, low height-for-age [<−2 (SD)] and normal height [−2 to +3 (SD)] were estimated. The measurements were being made weekly by nutritionists.

### 2.7. Dietary Assessment

A food frequency questionnaire (FFQ) was carried out, as well as a 24-h dietary recall (24 DR) [34]. The quantification of the average energy consumption (kcal), proteins (g), carbohydrates (g), fats (g), sugars (g), fiber (g), vitamin A (μg), B1 (mg), B2 (mg), B6 (mg), B12 (μg), C (mg), folic acid (μg), niacin (mg), vitamin E (mg), Ca (mg), Fe (mg), K (mg), Mg (mg), Na (mg), P (mg), Se (μg), and Zn (mg) were determined with NutriKcal^®^ (nutritional software based on food composition tables in Mexico and derived from 24-h dietary recall); on the other hand, adherence questionnaires and treatment monitoring were applied. The register was made and monitored weekly by nutritionists.

### 2.8. Hemoglobin Concentration

The concentration of hemoglobin (Hb) in capillary blood was carried out by photometry; participant was stung on a finger with a sterile lancet and Hb was measured from a drop of blood (preferably the third drop) using a microcuvette and deposited in the portable Hb analyzer (HemoCue^®^, 201, Brea, CA, USA), with a detection range of 0 to 24.6 g/dL. Hb was used to evaluate the absorption of nutrients from the diet during the intervention. The results were expressed as grams of hemoglobin per deciliter (g Hb/dL).

### 2.9. Urine Malondialdehyde Concentration

According to the most recent study carried out in the rural community of Valencianita where the first effects of a supplement were reported in the first month [28], it was decided to include 3 measurements, taking into account a Baseline measurement (week 0), at week 2 and at the end of the study (week 4).

The concentration of malondialdehyde in urine (UMDA) was carried out with a TBARS assay kit purchased from Cayman Chemical (Cayman Chemical, Ann Arbor, MI, USA) and according to the recommendations described by the manufacturer. Briefly, an aliquot of the urine sample (100 μL) was mixed with SDS solution (100 μL) and color reagent (4 mL) in a screw cap tube glass, and allowed to stand in a water bath at constant temperature (90 °C) for 1 h; later, tubes were removed and taken to an ice bath for 10 min to stop the reaction. After, samples were poured into 15 mL tubes to centrifuge for 10 min at 1600× *g* at 4 °C. Finally, 150 μL aliquots of the supernatant (duplicate) were taken and placed on clear plates (96-well solid plate) and read at an absorbance of 530–540 nm in a plate reader (Biotek instruments, ELx800, VT, USA). The concentration of the lipid peroxidation product was calculated from a standard MDA curve. The results were expressed as micromole of malondialdehyde per gram of creatinine (µM MDA/g creatinine).

### 2.10. Concentrations of Arsenic in Drinking Water and Urine

#### 2.10.1. Water

The concentration of arsenic (As) in drinking water was carried out using the hydrides generation technique using an As analysis kit (Arsenator^®^-Digital Arsenic Test Kit from Wagtech WTD, Palintest, CO, USA) for rural communities. The detection range of the Arsenator^®^ was 0 to 100 μg/L. Concentrations were expressed as micrograms of arsenic per liter (µg As/L). The evaluations were conducted weekly, one month prior to the study and during the intervention. School and household water sources used for consumption were analyzed. The procedure is described as follows: a 50 mL sample of water was obtained which was taken with a 250 mL flask, then a powder sachet (sulfamic acid) was added to the sample and a catalyst tablet, both provided by the kit. Then, two filter slides were used to remove (red) the excess of arsine gas and collect the arsenic gas (black) both inserted in a bung device. Immediately the bung device was pushed down firmly into the flask. Later, after 20 min, the black filter slide was removed from the bung device. The black arsenic filter slide was read into the DigiPAsS. The validation process was carried out according to the reference coloration parameters (Palintest).

#### 2.10.2. Urine

Quantification of As in urine (UAs) was performed according to the Cox method [35]. A 5 mL of urine sample was digested with a mixture of HNO_3_ and HClO_4_ (1:6); the mixture was stirred and covered with a watch glass and placed on a low-temperature hot plate for 15 min and later the temperature was increased to 80 °C until the sample was dried; briefly, the content was resuspended in 10 mL of HCl (3%). All procedures were performed in duplicate. For the reduction of As^+5^ to As^+3^, 5 mL of the digested sample was reduced with 1 mL of KI (3%) and 4 mL of HCl (1.5%) for 5 min at 80 °C in darkness. Before the process of reduction of the samples, the reagents for the hydrides generation were prepared; NaOH (1%) was prepared using deionized water, and later, NaBH_4_ (3%) was prepared with the NaOH solution previously made. Reagents were made separately and then mixed. The final NaBH_4_ solution was filtered using a #5 filter paper and a vacuum pump. The filtered solution was covered with aluminum foil and refrigerated for use on the same day. Finally, 10 mL of reduced sample was poured into a reaction flask; an average of 40 mL of NaBH_4_ reagent for every 6 samples analyzed was used. The sample was analyzed by atomic absorption spectrophotometry (AAS) with hydrides generation system (PerkinElmer, PinAAcle900H, Wellesley, MA, USA) using the software Syngistix 2.0 and under the following conditions: the wavelength of 197.20 nm, slit 0.7, EDL lamp (energy >50), argon as the carrier gas, and as signal mode the peak height. The results were expressed as micrograms of arsenic per gram of creatinine (µg As/g creatinine).

The concentration of As was calculated from a standard curve. The curve was calculated from a standard stock solution of 1000 ppb of As, dilutions were made for 0.5, 1.0, 5.0, 10.0, and 20.0 ppb of As, as well as a blank. The slope of the resulting curve was m = 0.0135 with a correlation coefficient of R^2^ = 0.9844.

For quality control, the ClinChek^®^ brand was used as a reference standard, with an average As concentration of 83.3 μg/L (66.6–99.9). For the preparation, 10 mL of deionized water was added to the control urine, and then 3 mL was taken and the digestion and reduction process were the same as the urine samples. A total of eight quality controls were performed with an average recovery percentage of 102.33% ± 2.43%.

The analysis considered 2 blanks (acids) and a quality control (ClinChek^®^) in duplicate for each batch (intervention week) evaluated.

### 2.11. Statistical Analysis

For the statistical analysis, the data were grouped according to age ranges, sex, and by group of participants (supplemented and controls). They were analyzed and compared using the statistical software SPSS^®^ 25.0. For selection of statistical tests, the Shapiro–Wilk normality test and Levene test for equality of variances were used. Comparative analysis between sex and study group was determined with the independent samples t Student and the Mann–Whitney U test for data with normality and without it respectively. The comparison of baseline and final variables was performed with paired sample t-Test and Wilcoxon signed-rank test; besides, to compare the intervention weeks, repeated measures ANOVA test was used as well as Friedman test, according to the characteristics of the variables. A *p* < 0.05 was used. To know the effect of the treatment the number needed to treat was calculated (NNT).

## 3. Results

### 3.1. Study Population

The groups formed were two, the first referred to as supplemented and the second one referred to as control, which were previously described. The indexes (height-for-age and body mass index for age) were calculated using the Z-Score for individuals ages 5–19 years. The baseline characteristics of both groups (supplemented and control group) were homogeneous (Table 1), without statistically significant differences between them (NS); only the percentage of fat mass and muscle mass showed differences, being higher in women and men respectively (Table 2).

### 3.2. Diet

Baseline analysis of the ingested nutrients is shown in Table 3 and Table 4. Evaluations showed scarce differences between supplemented and control group pre and post-intervention, showing that the dietary patterns remained homogeneous throughout the study. There was a significant difference by group (Table 3) only in energy (*p* < 0.002) and K (*p* < 0.05) intake; however the intake by sex (Table 4), showed that the macro and micronutrients were higher in men than in women (*p* < 0.002), with the exception of vitamin C, E, and magnesium where no significant difference was found; on the other hand, the intake of fiber and sugar did not show significant differences by sex.

Regarding the dietary reference intakes by age (9–13 years) for each macro and micronutrient [36], it was observed that the values for protein were according to the DRI (Dietary Reference Intakes) for men and women (>34 g/day); however, the intake of total fats and carbohydrates were high in both sexes. On the other hand, the men’s diet complied with the DRI for B vitamins, vitamin A, C, and niacin, but not for folic acid (78%) and vitamin E (24%). Regarding minerals, men showed adequate DRI for Fe, Mg, and Se, but the minimum recommended for Ca (85%), K (45%), P (60%), and Zn (75%) was not met.

High consumption of Na was reported for men (>2 g/day). On the other hand, women did not meet the minimum requirements for vitamins (16%–66%) and minerals (25%–77%) and regarding the sodium intake, did not exceed the recommended DRI (DRI: <2 g/day). Finally, according to the recommended dietary allowance of fiber, the intake was low in both sexes (DRI: 26–31 g/day).

On the other hand, according to the FFQ, the results showed a high intake (4–5 days per week) of ultra-processed food products (chips and pastries), sweets, popsicles, chocolates, cola drinks (Coke and Pepsi), and other carbonated beverages with flavorings, sweeteners, and high sugar content. Regarding meals provided at home, a preference for fried or oil foods was shown, mostly using this method and rarely roasted and steamed. Moreover, the consumption of fruits and vegetables was low, and the foods of greater preference by the participants were the banana, apple, and orange for fruits, and potatoes, onions, and tomatoes for vegetables (cooked), these with weekly consumption frequency (2–3 fruits per week; 2–4 vegetables per week); it is important to comment that the participants showed no interest in consuming raw (salad) or steamed vegetables (preference for fried and sugary foods), or as a snack due the bitter and weird flavors of these foods. Regarding animal products such as poultry and meat (beef and pork), the limitation of their frequency (1–2 times every 15 days) was observed due to costs, the egg and bean being the main source of protein (2–3 times per week), complemented with other foods, such as corn tortilla (daily) and pork rinds (1–2 times per week) to increase satiety.

These results show high consumption of ultra-processed foods, rich in sugars and saturated fats and low consumption of fruits and vegetables which reflects in the increase of carbohydrates, total fats, and sodium (Na) in the participants’ diet.

### 3.3. Hb Concentration

**Figure 2:**
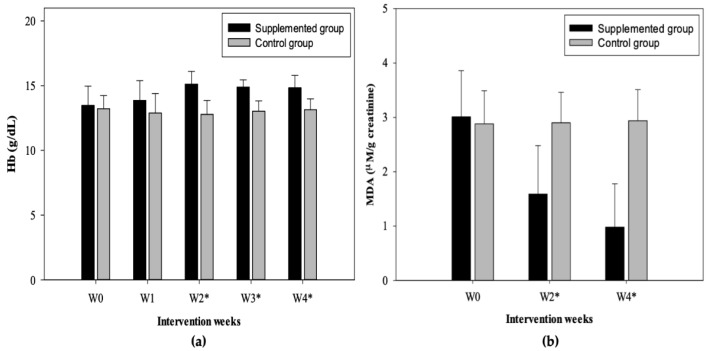
Concentrations of (**a**) Hb (Hemoglobin) (mean ± SD) and (**b**) UMDA (Urinary malondialdehyde) (mean ± SD) before, during, and after 4 weeks of supplementation in the supplemented and control group (W0: baseline; W1: week 1; W2: week 2; W3: week 3; W4: week 4). * Statistically significant difference between groups according to the independent samples t-Test (*p* < 0.001).

The Hb levels were compared, finding differences between both study groups (supplemented and control) (Figure 2a). The initial Hb averages were 13.47 ± 1.50 and 13.22 ± 1.01 g/dL for supplemented and control respectively; there were no differences in the baseline measurement and week 1, however, week 2, 3, and 4 showed statistically significant differences between supplemented group (W2: 15.12 ± 0.99; W3: 14.89 ± 0.56; W4: 14.83 ± 0.96 g/dL) and control (W2: 12.78 ± 1.08; W3: 13.02 ± 0.80; W4: 13.13 ± 0.85 g/dL) according to the independent samples t-Test (*p* < 0.001) (Figure 2a); reflecting an increase in this nutritional biomarker in the supplemented group from the second week of supplementation and later showing stable values in weeks 3 and 4.

Furthermore, a comparative analysis was performed between the baseline values and values obtained at the fourth week by the group of study (supplemented and control), which show statistically significant differences in the supplemented group (W0 vs. W4) according to the paired sample t-Test (*p* < 0.05); no differences were found for the control group.

### 3.4. MDA Concentration

According to the results of this research, no differences were found by intragroup (supplemented) sex; however, it was presented for the control group according to the independent samples t-Test (*p* < 0.05) (Table 5). On the other hand, significant differences were found between men of the supplemented and control group and women of the supplemented and control group after two weeks of intervention (Table 6). Comparative analysis between the supplemented and control group was performed, finding statistically significant differences (Figure 2b); in addition, a significant difference was found in the means of the concentration of MDA before (week 0) and after (week 4) of the supplementation (supplemented group) according to the paired sample t-Test (*p* < 0.001); on the other hand, according to the repeated measures ANOVA test, it was observed that the levels of MDA were different throughout the treatment, rejecting the equality of the means between weeks, and showing a decrease of this compound from week 2 (*p* < 0.001); the control group did not show significant differences between weeks. The Bonferroni correction was used, confirming the results previously mentioned.

### 3.5. As Concentrations in Water and Urine

A month prior to the study, a questionnaire was applied to know the main source of water for daily intake and food preparation; later, water was monitored in the school and participants’ homes (2 months), where the concentrations of As were identified. It was observed that 16% of the participants exclusively used purified water (bottle) to drink and prepare food, 15% reported consuming only drinking water to drink and prepare food, including water from the school tap during recess hours; the percentage of households that mixed water (purified water for drinking and household drinking water for food preparation) was 69%. The source and concentrations obtained of As in water samples were as follows: >100 ± 1.51 µg/L (drinking water from the school tap), 84.79 ± 17.00 µg/L (drinking water from the participants’ homes) and 1.94 ± 1.73 µg/L (purified water). The values were stable the prior month and during the study.

Regarding the concentrations of urinary As, Table 7 shows the values obtained by the group, sex, and week of intervention. The results showing a lack of significant differences in the supplemented group; however, in the control group, only a difference was shown by sex at week 3, according to the Mann–Whitney U test (*p* < 0.05).

**Figure 3:**
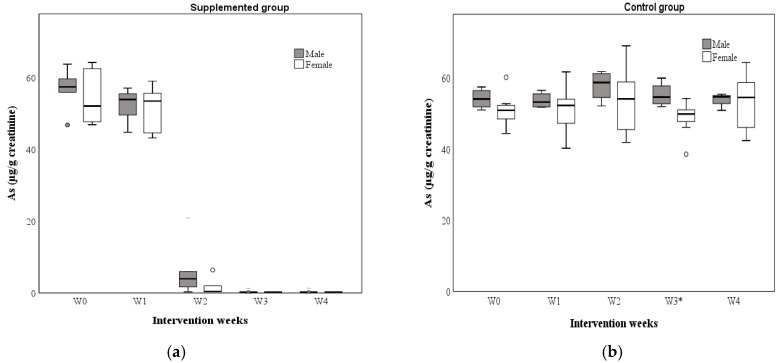
UAs (Urinary arsenic) (median) concentration by sex in the supplemented group (**a**) and control group (**b**) (W0: baseline; W1: week 1; W2: week 2; W3: week 3; W4: week 4). * Statistically significant difference by sex according to the Mann–Whitney U test (*p* < 0.05).

**Figure 4:**
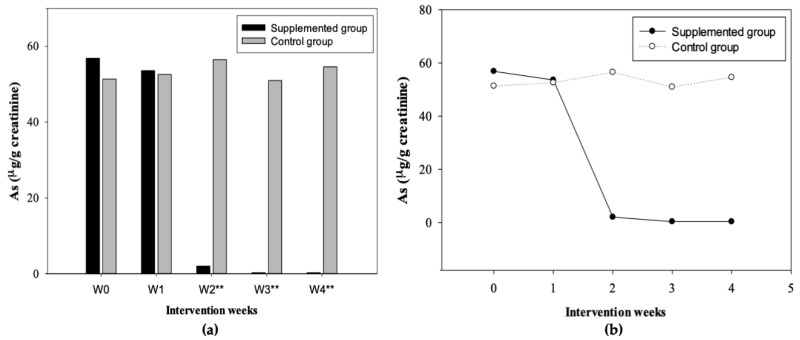
(**a**) Comparison of UAs (Urinary arsenic) concentrations (median) in the supplemented group and control group (W0: baseline; W1: week 1; W2: week 2; W3: week 3; W4: week 4). ** Statistically significant difference between groups according to the Mann–Whitney U test (*p* < 0.001); (**b**) Comparison of UAs concentration (median) between supplemented and control group per week of treatment. The Friedman and Wilcoxon tests were carried out to determine differences between weeks of supplementation.

In addition, Figure 3 shows the maximum and minimum UAs values obtained by sex in the supplemented (Figure 3a) and control (Figure 3b) group during 4 weeks of supplementation. On the other hand, the comparative analysis between the supplemented and control group was carried out, showing statistically significant differences at week 2, 3, and 4 (*p* < 0.001) (Figure 4a); also, according to the Wilcoxon signed-rank test, significant differences were found between weeks of supplementation (supplemented group) (Figure 4b). Differences were observed between most of the weeks (W0 vs. W1: *p* < 0.05; W1 vs. W2: *p* < 0.001; W2 vs. W3: *p* < 0.001; W0 vs. W4: *p* < 0.001), but without differences between W3 vs. W4 (NS) in the supplemented group; the control group did not show significant differences. Finally, the values of the two study groups before and after the intervention (W0-W4) were compared according to the Friedman test [*p*-value < 0.001; x2 (2) = 58.13]. The analysis reflected differences between the weeks of supplementation (supplemented group). In addition, the UAs medians obtained before (baseline: 56.85 µg/g creatinine) and after (W4: 0.29 µg/g creatinine) indicate treatment effectiveness by decreasing the concentrations of UAs through the time. The control group did not show significant differences [x2 (2) = 4.44, NS] (Figure 4b).

### 3.6. NNT

The number needed to treat for second week was NNT = 2 (CI = 2 to 4), RR = 4.47 (CI = 1.47 to 16.0), which indicates that to increase the urinary arsenic excretion, treating at least two is required.

## 4. Discussion

### 4.1. As in Drinking Water

Previous studies have reported high values of As in drinking water of this population [28], which when compared with the results found in the present study, have remained unchanged through the time; the present concentrations of this element remain of concern, as they exceed the established limits [29]. This element has been recognized as one of the most serious pollutants found in drinking water, which, in addition to being ubiquitous in the environment, has a wide list of adverse health effects [9].

Although tap water samples from school and homes contain concentrations up to 3 times higher than allowed (As: >100 µg/L) of this pollutant, which has been reported through communication programs in the same community [28], the changes observed in this study have been minimal since the percentage that continues to drink drinking water is higher (84%) compared to the participants who use exclusively purified water (16%). These findings can be attributed to the fact that there is a greater proportion of families that cannot afford to supply purified water, so that drinking water is used for all needs, including water for food consumption and preparation; according to Meza-Lozano et al. [37], families having no access to an economical source of purified water, prefer not to consume it because of the high cost involved. Therefore, it is necessary to propose strategies that, in addition to understanding direct communication, and water treatment, incorporate accessible food sources (*quelites*) rich in phytochemicals, which can be incorporated into the diet in the form of mixtures or formulations, since, at the moment, there are no changes and the exposure remains.

### 4.2. Hb, UMDA, and UAs

#### 4.2.1. Hb Concentration

The levels of Hb found in baseline were normal for men and women [38], however, this study showed that by incorporating a supplement with *quelites* into the diet, this nutritional biomarker can increase to 1.65 g/dL, concentrations that were higher compared to other studies. According to Egbi et al. [39] in a supplementation study with children ages 6–9 years, it was found that the consumption of green leafy vegetables, among them, plants of the genera *Amaranthus* spp., increases Hb concentrations in supplemented children (12.1 g/dL) compared to the control group (11.3 g/dL) at the end of the study, minimizing the prevalence of anemia (supplemented: 33.3%; control: 57.5%) in participants treated with the powder preparation. On the other hand, Monroy-Torres et al. [28] show that after supplementation in adolescents ages 12–15 years using a trademark based on grape, broccoli, and cranberry, Hb values increased 1 g/dL.

In this research, although both groups did not show low Hb levels, there was a significant difference from week 2 until the end of supplementation (Figure 2a). According to Egbi et al. [39], micronutrients such as Zn, Fe, and β-carotene from wild plant sources (green leafy vegetable powder), lead to an improvement in nutritional status, increasing Hb values and decreasing the proportions of anemia. Concentrations of this indicator are attributable to the quality of the diet, mainly the less varied diets with low consumption of fruits and vegetables, and mostly made up of cereals and legumes, have high concentrations of anti-nutrients such as tannins and phytates, known as inhibitors of the absorption and bioavailability of iron and zinc [39]. Another factor involved is inflammation processes, which affects red blood cells and retinol serum levels [39]. In addition, You et al. [40] report that fat consumption improves the bioavailability of carotene.

All these dietary patterns were observed in both study groups (Table 3 and Table 4). Therefore, the results obtained in the supplemented group show that supplementation with *quelites* provides essential micronutrients and possibly improves their bioavailability for the increase in Hb.

#### 4.2.2. MDA Concentration

MDA is a product of lipid peroxidation (LPO), the elevation of this in urine reflects the degree of oxidative stress, so this biomarker is usually used to evaluate LPO and DNA damage caused by exogenous free radicals or endogenous reactive oxygen species (ROS) [41]. It has been reported that the increase in MDA has a high correlation with exposure to pollutants, including As [41]. The results of MDA in both study groups (supplemented: 3.01 µM/g creatinine; control: 2.88 µM/g creatinine) (baseline), confirm the ability of this element to induce significant increases in MDA (Figure 2b; Table 5 and Table 6), indicating that participants have a high level of oxidative stress due to prolonged exposure. These values are similar to those reported by Wang et al. [41] which shows concentrations of 3.52 and 2.48 µM/g creatinine in men and women respectively (<20 years) exposed to As. Flora et al. [42] explain that the toxicity of As can be manifested directly with the attack on sulfhydryl groups or indirectly through the generation of ROS such as hydrogen peroxide, hydroxyl radical species, or superoxide anion, and where hydroxyl radicals play a role as initiators of LPO. On the other hand, according to Khuda-Bukhsh et al. [43], free radicals are electrophilic species that can react with cellular components. LPO process is initiated by the attack of a free radical, which could be emanated by As over unsaturated fats and the resulting chain reaction is terminated by the production of fat decomposition products such as alcohols and aldehydes (malondialdehyde).

On the other hand, in this study, after 4-week supplementation, the results showed a significant decrease in MDA concentrations (>50%) (Figure 2b; Table 5 and Table 6). Similar results have been reported in rats induced with toxic agents [23,27]; in these trials, it was revealed that when administered varying doses of *Chenopodium* spp. and *Portulaca* spp. plants, the production of hydrogen peroxide (40%) and concentration of MDA (50%) can be reduced; in addition, an increase in the activity of antioxidant enzymes such as superoxide dismutase (SOD), catalase (CAT), and glutathione peroxidase (GPX) was reported.

Some studies show that these antioxidant enzymes can reduce the toxic effects of ROS by eliminating them since they are the first line of defense against oxidative stress, however, these can be affected if ROS production is excessive, so phytonutrients become alternatives to minimize the toxic effect of As [42,44]. It has been reported that the presence of non-enzymatic antioxidants from food (plants, vegetables, leaves, flowers) helps to neutralize or remove free radicals [42,44]. These act by reducing peroxide concentrations and even inhibiting lipid peroxidation and repairing oxidized membranes or promoting the strengthening or restoration of the antioxidant defenses of the cells; on the other hand, supplementation with non-enzymatic antioxidant molecules (phytonutrients) reduce the possibility of the metalloid interacting with biomolecules and inducing oxidative damage [42,44]. Among these compounds are some phytochemicals of the phenolic type such as phenolic acids (caffeic acid), flavonoids (quercetin, naringenin, phloretin), and non-phenolic such as carotenoids (β-carotene), chlorophylls, vitamin A, vitamin E, vitamin C, some minerals like zinc, manganese, magnesium, and selenium and amino acids like cysteine [42,44,45], nutritional compounds that were presented in high concentrations in the supplement administered [30].

In addition, according to Pace et al. [44], the ability of phenolic antioxidants to neutralize oxidants is due to the donation of a hydrogen atom from polyphenolic antioxidants, leading to the formation of phenoxy-radical, which can be stabilized through intermolecular bonds between two polyphenols or by reaction with other radicals. Therefore, the results obtained in both groups are consistent with the reported evidence.

#### 4.2.3. UAs Concentration

Regarding UAs concentrations, the results showed that both groups had high baseline values with 51.34 µg/g creatinine and 56.85 µg/g creatinine for control and supplemented group respectively. The CDC [46] establishes diagnostic criteria for As, with concentrations greater than 50 µg/L, however, the health effects may vary depending on the acute or chronic exposure of this pollutant [9]. Subclinical complications were not evaluated in this research so their presence is not ruled out.

According to Monroy-Torres et al. [28], after supplementation in a group of adolescents ages 12–15, an increase in the excretion of UAs in the second week (53.9 µg/g creatinine) and third week (51.1 µg/g creatinine) was shown. However, our study did not register an increase in the excretion of this pollutant between weeks. The baseline values obtained were high, and later at the end of each week during the supplementation, a decrease of these concentrations was observed in the supplemented group (Table 7; Figure 4b). On the other hand, the results observed in the control group showed no significant difference between weeks, showing concentrations greater than 50 µg/g creatinine during the study (Table 7; Figure 4b). The variations found in this research regarding the literature may be due to the fact that the highest percentage of excretion of UAs may have occurred in the first days of the first and second week after supplementation, but with higher intensity in this latter week. Therefore, we infer that because the sample collection was carried out at the end of each week and not daily, the results obtained reflect the levels of this pollutant after its maximum excretion; thus, promoting an efficient balance and recovery process in the supplemented group. Nowadays, there are no clinical trials that report the biological effect of herbal remedies or the use of *quelites* as alternative recovery therapies in cases of arsenic exposure. Therefore, this research has reported interesting results. Our results showed an increase of UAs content during the first 2 weeks (high excretion and then a recovery process), and after 30 days of treatment, showing that a supplementation with *quelites* could enhance the mobilization of As during the first 15 days, observing a significant decrease (90%) of this pollutant (recovery process) (Table 7). In our study, UAs concentrations showed a fluctuation after the first week of treatment in the treated group and a notable difference being observed at week 2, resulting in an evident and periodic removal of arsenic from the body in the supplemented group. On the other hand, a decrease in the MDA level (50%) was observed (Figure 2b; Table 5 and Table 6). Other investigations have been reported similar behaviors to what was observed in our study about arsenic excretion. An intervention study [47] showed that in participants aged 18, urine arsenic concentrations were stable during the day, however, variations may exist throughout the days. Hence, the evidence of our study proposes a mechanism for the excretion of As in the urine and could establish a new balance process for this pollutant.

Regarding results obtained by sex, in our research, similar values of UAs among men and women without a statistical difference (Table 7) were observed. The literature reports an absence of differences in UAs concentrations by gender [47,48]. On the other hand, the significant decrease observed in this study is attributed to the fact that growing children have a more efficient process of methylation than adults because in this latter group, the number of factors that affect methylation increases with age, such as smoking, or decreased hepatic functions [48,49,50]. In addition, it has been reported that children retain lower amounts of As in the body, because the second step of the methylation process is more active and allows an increase in the value of concentrations [51]; so that the results obtained in this investigation are consistent and supported by the previously reported evidence, since the supplemented group showed a surprising reduction of this pollutant after supplementation (98%). On the other hand, As can also have different excretion patterns in participants of the same group (intra-individual variability; inter-individual variability) [52] or between groups [28,51]. However, the literature reports that many factors influence arsenic metabolism and condition its efficiency, including genetic factors (example: polymorphisms of the arsenite methyltransferase), metabolic capacity, sex, hormonal mechanisms, nutritional status, and diet [28,48,52,53].

During this clinical intervention study, it was observed that the diet of the participants in both study groups remained unchanged throughout the research. Despite the fact that the DRI of some macro and micronutrients were mainly met by men, most of these were from ultra-processed products, so the diet was considered of low quality in both groups due to the high consumption of these foods, such as drinks of cola and flavored drinks, foods high in saturated fats, and with high energy content, and on the other hand, a low consumption of plant-based foods was observed. According to Monroy-Torres et al. [54], it was observed that nutrient consumption considered as antioxidants in a population exposed to As was low, while the consumption of foods that promote greater oxidation and inflammation in the body was high (sugary drinks and ultra-processed foods), showing a high baseline concentration of As. This evidence is similar to that observed in our study for both groups, regarding the diet quality and the situation of the baseline concentrations of the biomarkers evaluated, and on the other hand, the behavior of biomarkers in the control group during the research (Table 3 and Table 4; Figure 2b and Figure 4a). According to Monroy-Torres et al. [54], this dietary situation promotes the onset of obesity and pathologies directly associated with arsenic (hepatopathy and nephropathy), directly contributing to the disorders and effects caused by exposure to the metalloid.

On the other hand, according to Hervert-Hernández et al. [55], the inclusion of fruits, vegetables, even roots, stems, and leaves contributes to high consumption of antioxidants, and therefore improves to the diet quality. However, nowadays the importance of consuming these potential sources of phytochemicals such as *quelites* has been forgotten, which could be used as alternative therapies to reduce the effects of various pathophysiological conditions, including As toxicity [21]. Some studies on populations exposed to As have reported that the consumption of natural supplements or foods rich in bioactive compounds improve the processes of methylation and increase the levels of excretion of As, promoting an efficient recovery process [28,42,53,56]. On the other hand, nutritional deficiencies due to low consumption of micronutrients contribute to increasing the risk of arsenic-induced skin lesions [57]. In our study, the *quelites* included in the supplement were evaluated (each plant and mixtures) showing an excellent nutritional profile when an optimized mixture was made [22,30]. The supplement was rich in antioxidant compounds [carotenoids, chlorophyll, phenolic acids (chlorogenic acid), flavonoids (quercetin, naringenin, phloretin, and phloridzin), amino acids (methionine, cysteine, serine, and glycine), minerals (Zn, Se, Mg, and P between others), and dietary fiber [22,30].

It has been reported that the administration of vitamin C leads to the formation of complexes with heavy metals and in addition with vitamin E, promotes an efficient recovery process by decreasing As concentrations [41]. On the other hand, supplementation with essential elements such as Zn and Se, reduces the toxicity effects of As, promoting their elimination, by chelation mechanisms, with the formation of reversible compounds [42,58]. Regarding Se, an antagonistic relationship with arsenic has been demonstrated where one reduces the toxicity of the other [59]; other studies report that possibly As and Se form a complex in the lysosome (As_2_Se) which is excreted in the urine [60]; in addition, As and Se compete for the union with functional proteins, thus reducing the availability of toxic metals [42]. Other studies have reported that the presence of selenium and folates increases the methylation of As in children, improving the efficiency of the 1-carbon metabolism, essential for the methylation and excretion of As. It is likely that this result in metabolism and the availability of methyl groups are particularly important in growing children to meet the production demands of creatine, proteins, phospholipids, and DNA [48].

On the other hand, other nutrients and compounds have been reported as beneficial, such as dietary fiber, carotenoids, and organic acids present in parts of plants and vegetables which reduce the toxic effects of heavy metals, including As [21,28,42]. Moreover, another study showed that low diets in green leafy vegetables and micronutrients such as calcium, folates, and vitamin C increase the probability of presenting toxic effects characteristic of As [57]. According to López-Carrillo et al. [56], the consumption of methionine, vitamin C, B6, B12, Fe, and Zn plays an important role in the metabolism of As. Each component improves the elimination of this toxic, participating in the metabolism of 1-carbon as catalyst components or donors of methyl groups. On the other hand, other studies report that vitamin C, methionine, and cysteine participate in the processes of As detoxification, promoting chelation and facilitating its removal from the body [14,42]. Finally, according to Kurzius-Spencer et al. [53], some amino acids are associated with the 1-carbon pathway such as methionine, cysteine, serine, and glycine, promoting the As methylation process. Other mechanisms have been reported to explain the beneficial effects of these nutrients and the exposure of As. Clemente et al. [61] describe that the treatments based on Fe, cysteine, flavonoids (quercetin, catechin, epigallocatechin), Mg, P, fruit, and vegetable extracts of green leaves (rich in polyphenols), reduce the transport of As through the intestinal monolayer, decreasing its absorption. Polyphenols have been reported to modulate tight junctions affecting toxic transport (paracellular permeability). On the other hand, there is possibly a relationship between the content of dietary fiber in food, the presence of As, and the interaction with the intestinal microbial ecosystem, favoring the DF-As ligand and lowering the percentage of bioaccessibility; however, this bioaccessibility will be influenced by intestinal motility, nutritional status, and genotype [62].

This evidence reinforces our results obtained in the supplemented group. As previously mentioned, during supplementation, a decrease in UAs concentrations was observed in the supplemented group and without differences in the control group, despite the content consumed of some components important for arsenic detoxification (Se, vitamin A, vitamin C, B vitamins) being similar in both study groups. On the other hand, the foods included in the diet were mostly ultra-processed. In addition, it was observed that during the entire study, other essential nutrients such as dietary fiber, vitamin E, folates, and Zn did not meet the dietary reference intakes in the supplemented and control group. Therefore, this indicates that the supplement administered provided a high-quality source of micronutrients and antioxidant compounds in the treated group that possibly allowed the reduction of the bioavailability of As, promoted the chelation and methylation process, reduced toxic effects, and finally, promoted a balancing mechanism and a recovery process.

Regarding the result of NNT being necessary to treat the observed from the second week on, it is important to compare this result with that of Monroy-Torres et al. [28], where the NNT was 7 but non confidence interval, which may lead us to demonstrate that we would be facing an advancement not only of the effects of the key nutrients that participate in arsenic excretion, but in what should be the best methodology and its intervention time.

This study approached the effect of a supplementation with two endemic *quelites* on urinary excretion of arsenic in adolescents exposed to water contaminated with the metalloid in a community in the state of Guanajuato for four weeks, which seems to increase the excretion of arsenic from the second week compared to the control group, in addition to improving other nutritional variables. Mexico is rich in plant biodiversity, where the *quelites*, in addition to being rich in nutrients, showed that they can have a beneficial effect on the urinary excretion of the metalloid.

## 5. Conclusions

This study showed that during supplementation based on two *quelites*, there was a significant reduction in MDA (1.59 µM/g creatinine) and UAs (2.02 µg/g creatinine) from week two; in addition to an improvement in Hb levels (15.12 g/dL). The decrease in arsenic was kept until the end of the treatment. Therefore, the incorporation in the diet of these plants high in phytochemicals could be a viable alternative therapy, by increasing the intake of macronutrients, micronutrients, and antioxidant compounds essential for detoxification, thus providing a more efficient recovery process against this pollutant. Prevention and orientation campaigns regarding consumption of tap water and the importance of a healthy diet have been carried out in this community; however, the current results show the null action to this problem, even though previous studies have reported this situation. Another reason that has also been considered, is the lack of technological solutions available to society, so it is necessary for sustainable strategies that can improve the nutritional and health status of members of rural communities exposed to arsenic, such as incorporating food with beneficial effects on the excretion of this pollutant.

## Figures and Tables

**Table 1:** General characteristics of participants of the supplemented and control group.

Baseline Variables	Supplemented *n* = 15	Control *n* = 12	*p*-Value
Age (years)	11.80 ± 0.41	12 ± 0.36	^3^ NS
Weight (kg)	47.31 ± 10.54	48.95 ± 11.33	NS
Height (cm)	150.31 ± 6.74	153.30 ± 7.21	NS
^1^ H/A interpretation (Z-Score)			NS
Normal height	15 ± 82.19	12 ± 91.42	
^2^ BMI interpretation (Z-Score)			NS
Obesity	2 ± 10.14	2 ± 16.05	
Overweight	5 ± 33.67	3 ± 19.36	
Normal weight	8 ± 31.94	7 ± 35.02	
Fat mass (%)	21.92 ± 14.71	23.02 ± 14.07	NS
Muscular mass (%)	33.46 *±* 11.08	32.12 ± 8.95	NS
Waist circumference (cm)	71.19 ± 12.05	71.48 ± 9.44	NS
Abdominal circumference (cm)	76.28 ± 11.58	79.33 ± 10.27	NS

The table shows the mean ± standard deviation (SD) for each baseline variable and the *p*-value according to the independent samples t-Test (*p* < 0.05). ^1^ H/A: height-for-age [ages 5–19 years (Z-Score)]; ^2^ BMI: body mass index for age [ages 5–19 years (Z-Score)]; ^3^ NS: not significant.

**Table 2:** General characteristics for men, women, and total participants included in the supplementation study.

Baseline Variables	Total Population *n* = 27	Men *n* = 13	Women *n* = 14	*p*-Value
Age (years)	11.88 ± 0.32	11.84 ± 0.37	11.92 ± 0.26	^3^ NS
Weight (kg)	48.13 ± 10.72	48.07 ± 10.89	48.19 ± 10.98	NS
Height (cm)	151.78 ± 7.01	152.56 ± 7.28	151.00 ± 6.64	NS
^1^ H/A interpretation (Z-Score)				NS
Normal height	27 ± 98.42	13 ± 86.24	14 ± 93.37	
^2^ BMI interpretation (Z-Score)				NS
Obesity	4 ± 31.26	1 ± 9.32	3 ± 17.21	
Overweight	8 ± 35.74	5 ± 35.11	3 ± 21.17	
Normal weight	15 ± 42.31	8 ± 32.29	7 ± 35.18	
Fat mass (%)	22.46 ± 14.16	13.98 ± 12.64	30.32 ± 10.74	<0.001
Muscular mass (%)	32.80 ± 10.03	40.47 *±* 9.25	25.67 ± 2.93	<0.001
Waist circumference (cm)	71.34 ± 10.77	71.20 ± 11.77	71.47 ± 10.20	NS
Abdominal circumference (cm)	77.81 ± 10.96	75.62 ± 10.90	79.84 ± 11.01	NS

The table shows the mean ± standard deviation (SD) for each baseline variable and the *p*-value according to the independent samples t-Test (*p* < 0.05). ^1^ H/A: height-for-age in men and women ages 5–19 years (Z-Score); ^2^ BMI: body mass index for age in men and women ages 5–19 years (Z-Score); ^3^ NS: not significant.

**Table 3:** Daily intake of energy and nutrients in supplemented and control group.

Nutrients	Supplemented *n* = 15	Control *n* = 12	*p*-Value
Energy (kcal)	2006.04 ± 5.07	2000.12 ± 3.49	<0.002
Protein (g)	70.05 ± 27.52	69.96 ± 27.96	^1^ NS
Fat (g)	70.02 ± 33.71	66.15 ± 29.66	NS
Cholesterol (mg)	284.21 ± 169.46	282.59 ± 180.64	NS
Carbohydrates (g)	290.38 ± 96.12	273.41 ± 51.34	NS
Sugar (g)	32.36 ± 31.67	30.02 ± 36.32	NS
Fiber (g)	21.09 ± 12.20	20.17 ± 8.91	NS
Vitamin A-Retinol (µg)	767.04 ± 182.09	753.62 ± 173.48	NS
Vitamin B1(mg)	1.15 ± 0.50	1.09 ± 0.38	NS
Vitamin B2 (mg)	1.23 ± 0.57	1.21 ± 0.45	NS
Vitamin B6 (mg)	0.94 ± 0.55	0.81 ± 0.46	NS
Vitamin B12 (µg)	1.93 ± 1.12	1.91 ± 1.27	NS
Vitamin C (mg)	52.47 ± 45.74	34.03 ± 29.79	NS
Folic acid (µg)	186.39 ± 174.18	136.25 ± 88.08	NS
Niacin (mg)	12.28 ± 6.60	9.08 ± 5.67	NS
Vitamin E (mg)	2.67 ± 2.01	1.97 ± 3.73	NS
Ca (mg)	947.59 ± 361.72	937.66 ± 363.81	NS
Fe (mg)	14.82 ± 7.30	13.29 ± 4.43	NS
K (mg)	1605.14 ± 654.99	1094.35 ± 566.09	<0.05
Mg (mg)	303.39 ± 220.35	240.26 ± 184.69	NS
Na (mg)	2501.90 ± 811.04	1949.78 ± 721.42	NS
P (mg)	631.40 ± 304.63	595.08 ± 264.43	NS
Se (µg)	51.95 ± 30.00	50.06 ± 23.06	NS
Zn (mg)	4.68 ± 2.60	4.26 ± 2.00	NS

The values show the mean ± SD for each nutrient and the *p*-value according to the independent samples *t*-Test (*p* < 0.05). ^1^ NS: not significant.

**Table 4:** Daily intake of energy and nutrients of all adolescents and by group (men and women) included in the supplementation study.

Nutrients	Total Population *n* = 27	Men *n* = 13	Women *n* = 14	*p*-Value
Energy (kcal)	2003.07 ± 1.24	2345.11 ± 1.35	1490 ± 1.11	<0.001
Protein (g)	70.24 ± 32.01	87.84 ± 27.96	43.85 ± 19.88	<0.001
Fat (g)	68.19 ± 29.36	81.03 ± 26.83	48.93 ± 8.62	<0.001
Cholesterol (mg)	283.63 ± 127.38	398.28 ± 142.17	111.67 ± 101.48	<0.001
Carbohydrates (g)	281.85 ± 82.19	321.01 ± 72.98	223.13 ± 27.13	<0.001
Sugar (g)	31.07 ± 29.70	38.01 ± 36.21	20.65 ± 14.98	^1^ NS
Fiber (g)	20.64 ± 8.56	22.81 ± 10.68	17.39 ± 3.81	NS
Vitamin A-Retinol (µg)	759.60 ± 128.11	877.67 ± 143.62	582.50 ± 105.69	<0.001
Vitamin B1(mg)	1.13 ± 0.54	1.41 ± 0.52	0.69 ± 0.11	<0.001
Vitamin B2 (mg)	1.22 ± 0.65	1.60 ± 0.55	0.64 ± 0.30	<0.001
Vitamin B6 (mg)	0.87 ± 0.56	1.11 ± 0.50	0.50 ± 0.34	<0.001
Vitamin B12 (µg)	1.92 ± 1.10	2.46 ± 1.01	1.11 ± 0.88	<0.001
Vitamin C (mg)	43.30 ± 44.13	52.41 ± 50.86	29.65 ± 11.81	NS
Folic acid (µg)	161.33 ± 198.05	235.89 ± 219.80	49.49 ± 21.16	<0.002
Niacin (mg)	10.69 ± 6.75	13.97 ± 6.61	5.76 ± 1.50	<0.001
Vitamin E (mg)	2.33 ± 1.80	2.67 ± 1.80	1.82 ± 1.87	NS
Ca (mg)	942.63 ± 270.27	1133.50 ± 332.51	656.33 ± 255.79	<0.001
Fe (mg)	14.10 ± 9.59	18.31 ± 9.57	7.79 ± 2.49	<0.001
K (mg)	1349.47 ± 436.12	1597.94 ± 445.58	976.75 ± 407.24	<0.001
Mg (mg)	271.83 ± 222.07	328.83 ± 238.09	186.33 ± 164.75	NS
Na (mg)	2226.77 ± 658.33	2993.61 ± 776.12	1076.50 ± 501.16	<0.001
P (mg)	613.67 ± 307.02	754 ± 220.09	403.17 ± 225.73	<0.001
Se (µg)	51.33 ± 29.33	67.56 ± 19.44	27 ± 14.33	<0.001
Zn (mg)	4.46 ± 2.94	5.99 ± 2.49	2.15 ± 1.37	<0.001

The values show the mean ± SD for each nutrient and the *p*-value according to the independent samples t-Test (*p* < 0.05). ^1^ NS: not significant.

**Table 5:** Urinary MDA concentrations in men, women, and total participants before, during, and after 4 weeks of supplementation.

	Supplement Total Participants *n* = 15	Men *n* = 9	Women *n* = 6		Control Total Participants *n* = 12	Men *n* = 4	Women *n* = 8	
Weeks	Urinary MDA concentrations (µM/g creatinine)							
				*p*-value				*p*-value
Baseline	3.01 ± 0.85	3.26 ± 0.97	2.63 ± 0.48	NS	2.88 ± 0.61	3.44 ± 0.35	2.59 ± 0.51	<0.014
2	1.59 ± 0.89	1.84 ± 1.06	1.21 ± 0.36	NS	2.90 ± 0.56	3.47 ± 0.39	2.61 ± 0.38	<0.004
4	0.98 ± 0.80	1.13 ± 1.01	0.75 ± 0.28	NS	2.94 ± 0.57	3.51 ± 0.26	2.66 ± 0.44	<0.006

The values show the mean ± SD of UMDA (Urinary malondialdehyde) concentrations (µM/g creatinine) in supplemented and the control group before, during and after 4 weeks of supplementation. The week zero represents baseline. NS: no statistically significant difference between men and women of the same group per week according to the independent samples *t*-Test.

**Table 6:** Urinary MDA concentrations in men of the supplemented and control group and women of the supplemented and control group, before, during, and after 4 weeks of supplementation.

	Men Supplemented *n* = 9	Control *n* = 4		Women Supplemented *n* = 6	Control *n* = 8	
Weeks	Urinary MDA concentrations (µM/g creatinine)					
			*p*-value			*p*-value
Baseline	3.26 ± 0.97	3.44 ± 0.35	NS	2.63 ± 0.48	2.59 ± 0.51	NS
2	1.84 ± 1.06	3.47 ± 0.39	<0.014	1.21 ± 0.36	2.61 ± 0.38	<0.001
4	1.13 ± 1.01	3.51 ± 0.26	<0.001	0.75 ± 0.28	2.66 ± 0.44	<0.001

The values show the mean ± SD of UMDA (Urinary malondialdehyde) concentrations (µM/g creatinine) in men of supplemented and control group and women of supplemented and control group, before, during and after 4 weeks of supplementation. The week zero represents baseline. NS: no statistically significant difference between men of supplemented and control group and women of supplemented and control group per week according to the independent samples *t*-Test.

**Table 7:** Urinary As concentrations in the supplemented and control group during 4 weeks of supplementation.

	Supplemented Total Participants *n* = 15	Men *n* = 9	Women *n* = 6		Control Total Participants *n* = 12	Men *n* = 4	Women *n* = 8	
Weeks	Urinary As concentrations (µg/g creatinine)							
				*p*-value				*p*-value
0	56.85	57.42	52.09	NS	51.34	53.98	50.81	NS
1	53.60	53.90	53.48	NS	52.57	53.13	52.19	NS
2	2.02	3.95	0.45	NS	56.49	58.62	54.02	NS
3	0.29	0.27	0.30	NS	50.97	54.53	49.76	<0.05
4	0.29	0.27	0.30	NS	54.60	54.78	54.45	NS

The values show the median of UAs (Urinary arsenic) concentrations (µg/g creatinine) in the supplemented and control group. The week zero represents baseline. NS: no statistically significant difference between men and women of the same group per week according to the Mann–Whitney U test.
